# Using Randomized Controlled Trials to Estimate the Effect of Community Interventions for Childhood Asthma

**DOI:** 10.5888/pcd20.220351

**Published:** 2023-06-01

**Authors:** W. Dana Flanders, Tursynbek A. Nurmagambetov, Cheryl R. Cornwell, Andrzej S. Kosinski, Kanta Sircar

**Affiliations:** 1 Rollins School of Public Health, Emory University, Atlanta, Georgia; 2 Division of Environmental Health Science and Practice, National Center for Environmental Health, Centers for Disease Control and Prevention, Atlanta, Georgia; 3 Oak Ridge Institute for Science and Education, Oakridge, Tennessee; 4 Department of Biostatistics and Bioinformatics, School of Medicine, Duke University, Durham, North Carolina

## Abstract

**Introduction:**

The Centers for Disease Control and Prevention’s Controlling Childhood Asthma and Reducing Emergencies initiative aims to prevent 500,000 emergency department (ED) visits and hospitalizations within 5 years among children with asthma through implementation of evidence-based interventions and policies. Methods are needed for calculating the anticipated effects of planned asthma programs and the estimated effects of existing asthma programs. We describe and illustrate a method of using results from randomized control trials (RCTs) to estimate changes in rates of adverse asthma events (AAEs) that result from expanding access to asthma interventions.

**Methods:**

We use counterfactual arguments to justify a formula for the expected number of AAEs prevented by a given intervention. This formula employs a current rate of AAEs, a measure of the increase in access to the intervention, and the rate ratio estimated in an RCT.

**Results:**

We justified a formula for estimating the effect of expanding access to asthma interventions. For example, if 20% of patients with asthma in a community with 20,540 annual asthma-related ED visits were offered asthma self-management education, ED visits would decrease by an estimated 1,643; and annual hospitalizations would decrease from 2,639 to 617.

**Conclusion:**

Our method draws on the best available evidence from RCTs to estimate effects on rates of AAEs in the community of interest that result from expanding access to asthma interventions.

**Summary**

**What is already known on this topic?**

It is critical to estimate the effect of expanding asthma interventions on the rates of adverse asthma events (AAEs). Public health programs use the results of randomized controlled trials (RCTs) in practical implementation for their population.

**What is added by this report?**

Consistent examination of translating results of RCTs into effectiveness outcomes has been limited. We describe and illustrate a method of using the results of RCTs to estimate changes in rates of AAEs and provide examples of how to apply the method.

**What are the implications for public health practice?**

This method can be used to calculate anticipated effects of future, planned interventions, or retrospectively, to calculate estimated the effects of already implemented programs. Public health practitioners can use the method outlined in this article and the current base of RCT literature (and the results of future RCT studies as they become available) in their planning and evaluation of interventions.

## Introduction

More than 4.2 million children in the US had current asthma in 2020; they had more than 0.79 million emergency department (ED) visits and 64,525 hospitalizations with a primary asthma diagnosis in 2019 (1,2). The health impact of pediatric asthma is accompanied by high costs (3). The Centers for Disease Control and Prevention’s (CDC’s) National Asthma Control Program developed and published the EXHALE Technical Package in 2018 (4). This resource comprises 6 evidence-based strategies that can be used to help people achieve better asthma control and decrease the number of asthma-related ED visits and hospitalizations (4). These 6 strategies are 1) education on asthma self-management (“E,” hereinafter referred to as E1), 2) extinguishing smoking and exposure to secondhand smoke (“X”), 3) home visits for trigger reduction and asthma self-management education (“H”), 4) achievement of guidelines-based medical management (“A”), 5) linkages and coordination of care across settings (“L”), and 6) environmental practices to reduce asthma triggers from indoor, outdoor, or occupational settings (“E,” hereinafter referred to as E2) (4). Implementing and expanding access to these strategies, in part by leveraging partnerships with public and private partners, is a current priority for National Asthma Control Program (4). In 2019, the Controlling Childhood Asthma and Reducing Emergencies initiative was launched with the goal of preventing 500,000 ED visits and hospitalizations nationwide within 5 years through implementation and expansion of access to EXHALE strategies in the population of children with asthma (5).

Evidence for effectiveness of EXHALE strategies differs from strategy to strategy in the type, number, and quality of relevant published studies (4). Four of these strategies — education on asthma self-management, extinguishing smoking, home visits for trigger reduction and asthma self-management education, and achieving guidelines-based medical management (6) — are implemented at an individual level (7). In contrast, the remaining 2 strategies — environmental policies or best practices to reduce asthma triggers from indoor, outdoor, and occupational sources and linkages and coordination of care across settings — address policies or regulations. Each strategy promotes various asthma interventions, which can be tailored to various groups of people with asthma (for example, by demographic and socioeconomic status, geographic location, place of intervention, and asthma severity) (4). For example, an asthma self-management education intervention could involve instructions on using medications correctly, recognizing and managing symptoms, and reducing exposure to asthma triggers and could take place in a school, clinic, home, or other setting. A full description of EXHALE strategies is available at www.cdc.gov/asthma/exhale/index.htm.

The primary objective of this study was to describe and illustrate a method of using results of randomized controlled trials (RCTs) to estimate changes in rates of adverse asthma events (AAEs) that result from expanding access to an EXHALE strategy. The method can also be applied to other study designs, such as cohort and case-control studies, if they generate valid effect estimates. It can be used to calculate anticipated effects of future Controlling Childhood Asthma and Reducing Emergencies interventions or estimate impacts of previously implemented programs. Expanding asthma interventions is a major public health approach to decreasing asthma-related illness and death, so it is important to estimate the effect of an expansion in access to these interventions on rates of AAEs.

## Methods

We illustrate a method for using the results of RCTs to estimate the effect of asthma-related interventions on reducing AAEs among children. We used counterfactual reasoning to justify a formula for estimating the expected number of AAEs prevented by implementing selected EXHALE strategies. Asthma interventions are commonly offered to only a part of the population with asthma. First, we show how to predict or estimate effects in this situation. Second, we use data extracted from relevant RCTs identified in the literature to illustrate an application of the formula. Finally, we discuss the approach, results, and considerations for when the method should apply.

### Selection of RCTs

We reviewed the literature to identify RCTs for each EXHALE strategy. We restricted the pool of eligible RCTs to those that were 1) included in any of 3 systematic reviews (8–10) or the EXHALE technical package (4), 2) recently published (in 2000 or later), 3) included children with asthma, and 4) were conducted in North America (11). We excluded studies for a given outcome if they reported pre-intervention rates of AAEs that differed between the treatment and control arms by more than 30%, indicating potentially imbalanced randomization (12–15). 

### Effects in the RCTs

In the RCTs considered, an EXHALE strategy was offered to all participants in the treatment arm but not to participants in the control arm, who generally continued to receive care as usual. Some participants in the treatment arm may not have complied with, taken advantage of, or used the intervention. All participants were followed during the RCTs to determine rates of AAEs. We denoted the observed rates of AAEs in the treatment arm as Rate_T_ and in the control arm as Rate_C_ and the rate ratio (RR) by

${RR}_{RCT_{E}}={Rate}_{T}/Rate_{C}$ (Equation 1)

where subscript E refers to EXHALE strategy E1 (education on asthma self-management), which is made available by the intervention. In a well-conducted RCT, we expect ${RR}_{RCT_{E}}$ to be a consistent estimator of the average causal effect of making E available to the study population (assumption 1, Table 1).

**Table 1:** Notation and Assumptions Used to Estimate the Effect of an Intervention on the Community

Notation	Explanation or comment
ΔEvents	Expected change in number of asthma adverse events; (a negative value indicates expected prevention of these events)
${RR}_{RCT_{E}}={Rate}_{T}/Rate_{C}$	Rate ratio comparing adverse event rate in treatment arm (Rate_T_) with control arm (Rate_C_)
Rate_p_	Adverse event rate in those offered the intervention in the community (the population of interest)
${Rate}_{\overset{-}{P}}$	Adverse event rate in those not offered the intervention in the community
$Rate_{\overset{-}{P}}^{*}$	Counterfactual adverse event rate among those in the community offered the intervention if (contrary to fact) they had not been offered it
**Assumptions**	Stated quantitatively
1. RCT is well-designed, conducted, and analyzed	${RR}_{RCT_{E}}$ estimates the causal effect of the program in the RCT (study population)
2. Asthma intervention offered to enrollees was similar to the intervention evaluated in the RCT	Relevance
3. The effect in the RCT validly estimates the effect among those in the community who were offered the program	${RR}_{RCT_{E}}=Rate_{P}/Rate_{\overset{-}{P}}^{*}$; if the baseline rate ratio for the comparison group vs the intervention group was available, then we used the ratio of rate ratios to correct for baseline differences
4a. Rate_p_ is observed	The rate in community enrollees directly observed
4b. $Rate_{\overset{-}{P}}^{*}={Rate}_{\overset{-}{P}}$	Counterfactual rate $Rate_{\overset{-}{P}}^{*}\;$ (if the intervention were *not* offered) can be estimated by an observed rate ${Rate}_{\overset{-}{P}}$, such as rates in those in the rest of the community (nonparticipants or those not offered the intervention)
4c. Can estimate number of events expected in the community if the program had not been implemented	If value for events can be estimated based on numbers in a prior year, then this number can be used in equation

Abbreviation: RCT, randomized control trial.

### Effect in a community where a subpopulation is selected for an intervention

We considered the effects of a particular intervention offered to only a part of a population of interest (for example, all children with asthma in a county). We assumed that the study population was not selected from the community and that only proportion (*Pr*) was enrolled to receive the intervention (enrollees). We denoted the rates of AAEs among enrollees as Rate_P_ and the rate in the community among those not enrolled (nonparticipants) as ${Rate}_{\overset{-}{P}}$. We assumed that the asthma intervention offered to the enrollees was similar to the intervention evaluated in the RCT (assumption 2, Table 1). The intervention is made available to all enrollees, but not everyone will necessarily use or take advantage of its availability. This situation parallels the situation in an RCT, in which some participants in the intervention arm may not take advantage of or use the intervention offered (ie, less than complete compliance). Furthermore, before or during a community program, the intervention may have already been available as part of usual care to nonparticipants in the community. Again, this situation parallels the situation of imperfect compliance in an RCT wherein some participants in the control group receive the intervention as part of usual care outside the study.

The key assumption is that the effect on community enrollees approximates the situation in the RCT with effects measured on the appropriate scale (assumption 3, Table 1 [16]). The approximation need hold only within strata of covariates if stratum-specific estimates are available. Here, we use the rate ratio (${RR}_{RCT_{E}}$) to measure effects. We equated the effect of the intervention in the RCT to that among community enrollees:

${RR}_{RCT_{E}}=Rate_{P}/Rate_{\overset{-}{P}}^{*}$ (Equation 2)

where $Rate_{\overset{-}{P}}^{*}$ is the counterfactual rate among community enrollees if they (contrary to fact) had not been enrolled in the intervention. Equation 2 directly parallels a counterfactual formulation of the causal effect in those enrolled to receive the intervention: it is the ratio of the rate if all were enrolled compared with the counterfactual rate if none were enrolled (17). Equation 2 encodes the assumption that the causal effect among community enrollees, measured as a rate ratio, equals the rate ratio in the RCT without further adjustment (16,18). This assumption is related to transportability, a concept that holds if the causal effect of treatment in the study sample applies to the target population that is distinct from that sample.

### Effect on adverse events among community enrollees

We derived expressions for the expected number of adverse events prevented, assuming that the program only affected enrollees. We let ΔEvents denote the expected change in the number of adverse events, in a community of size N in which the proportion *Pr* was enrolled in the program. We estimated the change in the annual number of events by the rate difference (rate per person per year if enrolled minus rate not enrolled) times the number enrolled, *PrN*:

$∆Events=PrN{(Rate}_{P}-Rate_{\overset{-}{P}}^{*})$ (Equation 3)

Equation 3 can be written in several different but equivalent ways to allow various applications according to which combination of measures (${Rate}_{P},\;Rate_{\overset{-}{P}}^{*},{RR}_{RCT_{E}},$ and $Events=N\cdot Rate_{\overset{-}{P}}^{*}$) is available and thought to be most suitable for the population of interest.

$∆Events=PrN{Rate}_{P}(1-{1/RR}_{RCT_{E}})$ (Equation 4a) or

$=PrN\left({RR}_{RCT_{E}}-1\right)Rate_{\overset{-}{P}}^{*}$ (Equation 4b) or

$=Pr\left({RR}_{RCT_{E}}-1\right)Events$ (Equation 4c)

where $Events=N\cdot Rate_{\overset{-}{P}}^{*}$ represents the expected number of events in the community, if the program had not been implemented. Which equation, 4a, 4b, or 4c, is best applicable depends on availability of additional information as described next.

If we observed the rate among community enrollees (Rate_p_) and have a suitable rate ratio ${(RR}_{RCT_{E}})$ from one or more RCTs, we can apply Equation 4a to estimate the expected change in the number of events.

If the rate among community enrollees (Rate_p_) is not available, it may be possible to estimate $Rate_{\overset{-}{P}}^{*}$, the rate among enrollees, if they had not been enrolled ($Rate_{\overset{-}{P}}^{*}$) and use an applicable rate ratio ${(RR}_{RCT_{E}})$ from an RCT in Equation 4b to estimate ΔEvents. For example, it may be reasonable to estimate $Rate_{\overset{-}{P}}^{*}$ as ${Rate}_{\overset{-}{P}}$, the rate observed in the remainder of the community (or a relevant subgroup) where the intervention was not implemented. It could be plausible to assume that the observed ${Rate}_{\overset{-}{P}}$ satisfies $Rate_{\overset{-}{P}}^{*}={Rate}_{\overset{-}{P}}$

where, ${Rate}_{\overset{-}{P}}$ denotes the (observed) rate in a subgroup or community that was not offered the intervention (Table 1, assumption 4b). This assumption should hold if the intervention had no effect on rates in parts of the community where it was not offered, and the enrollees were comparable to the rest of the community with respect to rates. In this case, the estimate of $Rate_{\overset{-}{P}}^{*}$ and of ${RR}_{RCT_{E}}$, if applicable to the community of interest, allows application of Equation 4b. Alternatively, it may be reasonable to use observed rates in another community that is not offered the intervention as an estimate of $Rate_{\overset{-}{P}}^{*}$. Yet another alternative could be to use the observed rate among enrollees in the year before the intervention was introduced as an estimate of $Rate_{\overset{-}{P}}^{*}$. However, major disruptions in health and health care, such as a pandemic, can render this option inappropriate.

Alternatively, if the number of events expected in the community had the program not been implemented can be estimated, perhaps based on numbers in a prior year, then this number can be substituted for “events” in Equation 4c.

For completeness, if the rate among enrollees Rate_P_ is measured and a direct estimate of the rate among enrollees had they not enrolled $\left(Rate_{\overset{-}{P}}^{*}\right)$ is also available, then the number of adverse events prevented can be estimated by direct substitution into Equation 3. This approach, however, would not incorporate results of the RCTs.

### Implementation

Implementation of the approach for estimating the effect of asthma-related interventions on rates of AAEs involved the following steps. First, we reviewed the literature to identify relevant studies that provided a valid effect estimate for one or more interventions similar to the ones of interest (Assumptions 1 and 2, Table 1). We summarized characteristics of the identified studies, including the EXHALE strategy used, the type of intervention implemented, the demographic characteristics of the population studied (the number of participants, age group, percentage that was male, and race and ethnicity), the severity of asthma in the study population, the baseline rates in the hospital or ED or both, the study period, and the location of the study. Second, we evaluated each study’s population and compared its characteristics with those of the community to assess the extent to which the effect in the RCT might validly estimate the effect among those in the community who were offered the program (Assumption 3, Table 1, transportability). The most appropriate RCT to use depends on these characteristics, comparability of the study population with the target community, and distributions of effect modifiers. Third, we evaluated available information to identify which combination of information (${Rate}_{P},\;Rate_{\overset{-}{P}}^{*}$, or $Events=N\cdot Rate_{\overset{-}{P}}^{*}$) was available in addition to ${RR}_{RCT_{E}}$ (Assumption 4a, 4b, or 4c; Table 1). Finally, we calculated the number of prevented events by using Equations 4a, 4b, or 4c, where the choice was guided by the third step (evaluation of available information).

We developed 2 examples to illustrate how we implemented our approach. In Example 1, we considered the projected number of ED visits prevented by a community program if the program expanded access to a specific EXHALE strategy: E, A, X, or E + A + H + L. We first considered the number of ED visits prevented by expanding access to strategy E, education on asthma self-management. We separately estimated the effect of a program in each relevant RCT. We estimated $Rate_{\overset{-}{P}}^{*}$ by assuming it approximately equaled the observed rate in the community of interest during the year before the planned intervention. We calculated the anticipated effect of expanding this strategy by offering a program to *Pr* = 20% of a community with 20,540 asthma-related ED visits among children; we assumed this number of ED visits on the basis of publicly available data from Los Angeles County (19). Similarly, we used the same approach to calculate the number of ED visits prevented by programs expanding access to strategies A, X, and E + A + H + L.

In Example 2, we considered the projected number of hospitalizations prevented by a community program if the program expanded access to strategy E, A, or X. We first considered strategy E. To apply Equation 4b, we must choose a study in which the children with asthma studied in the RCT were sufficiently similar to those in the community population for the rate ratios to be applicable. Here, we estimated $Rate_{\overset{-}{P}}^{*}$ by assuming that it approximately equaled the observed rate in the community of interest in the year before the planned intervention. To predict the number of hospitalizations prevented, we again presumed that *Pr* was equal to 0.2 (ie, the program provided access to self-management education for 20% of the community population). On the basis of data from Los Angeles County (19), 2,639 asthma-related hospitalizations occurred in 2015 among children. Next, we applied the rate ratio (${RR}_{RCT_{E}}$ = 0.21) from the study by Bruzzese et al (20) and applied Equation 4c. For other studies, we similarly estimated the number of hospitalizations prevented when the rate ratio from the respective RCT was transportable to the population of interest.

## Results

Our literature search identified 15 RCTs meeting inclusion criteria (Table 2). Study populations ranged in size from 56 to 937. Participant characteristics differed from study to study. For example, asthma severity varied across the studies; 1 study included only children with severe asthma, while other studies included children with any asthma diagnosis. We found only 1 RCT for strategy X (29) and no RCTs met our selection criteria for strategy H or for strategy E2.

**Table 2:** Characteristics of RCT Studies of Children With Asthma Published Since 2000 in North America

Study	EXHALE strategy^a^	Intervention	Demographic characteristics	Asthma severity	Baseline rates^b^	Years of study	Location(s)
Atherly et al (12), 2009	Education on asthma self-management	School-based asthma education (three 90-min educational sessions)	N = 524; grades 6–12; 51% male; race and ethnicity not reported	Not reported	Hospital: control = 0.4, intervention = 0.3	2003–2004	Kansas City, Kansas; Stafford/Fredericksburg, Virginia
Bender et al (11), 2015	Achievement of guidelines-based medical management	Speech recognition telephone calls for inhaled corticosteroid refill	N = 899; aged 3–12 y; 64% male; 56% White, 14% Black, 25% Hispanic	Persistent asthma diagnosis	ED: control = 0.1, intervention = 0.1; Hospital: control = 0.04, Intervention = 0.04	2009–2013	Kaiser Permanente Colorado
Brown et al (21), 2006^c^	Education on asthma self-management	Asthma education delivered by trained asthma nurse-educator	N = 239; aged <18 y; 46% male; 30% Black, 59% White, 11% Other	56% Moderate to severe persistent	Not reported (ED)	2004	Community hospital in Grand Rapids, MI
Bruzzese et al (20), 2011	Education on asthma self-management	School-based intervention for asthma self-management	N = 345; grades 9–10; 30% male; 46% Hispanic, 38% Black, 16% Other	Moderate to severe persistent	ED: control = 1.9, intervention = 1.8; Hospital: control = 0.2, intervention = 0.2	2001–2004	New York City
Cabana and Thyne (22), 2006	Achievement of guidelines-based medical management	Continuing medical education program for physicians	N = 870; aged 2–12 y; 65% male; race and ethnicity not reported	37% Persistent	ED: control = 0.7, intervention = 0.9; Hospital: control = 0.1, intervention = 0.1	2001–2003	10 US regions
Cowie et al (23), 2002	Education on asthma self-management	Education on asthma management	N = 62; aged 15–20 y; 29% male; race and ethnicity not reported	Severe	ED: control = 3.0, intervention = 3.2; Hospital: control = 0.3, intervention = 0.2	Not reported	Calgary, Alberta
Farber et al (24), 2004	Education on asthma self-management	Asthma education provided in emergency department	N = 56; aged 2–18 y; sex not reported; predominantly Black	Poorly controlled	Not reported (hospital or ED)	Not reported	New Orleans, Louisiana
Gorelick et al (25), 2006	Education on asthma self-management	Primary care linkage and initiation of asthma case management	N = 352; aged 2–17 y; 66% male; 69% Black, 21% White, 8% Latino	70% Persistent	Not reported (ED)	2003–2004	Children’s Hospital of Wisconsin, Milwaukee, Wisconsin
Halterman et al (26), 2004	Achievement of guidelines-based medical management	School-based care	N = 180; aged 3–7 y; 63% male; 59% Black, 12% White, 32% Hispanic, 29% Other	Mild persistent to severe persistent	Hospital: control = 0.3, intervention = 0.5	2000–2002	Rochester, New York
Harish et al (13), 2001	Education on asthma self-management	Asthma care in specialty clinic (3 visits including medical and environmental control, education, close monitoring, and 24-h availability)	N = 129; aged 2–17; sex not reported; majority Hispanic and Black	Not reported (patients treated at pediatric ED)	Hospital: control = 0.4, intervention = 0.4	1994–1995	Bronx Lebanon Hospital, South Bronx, New York
Horner et al (14), 2016	Education on asthma self-management	School-based asthma class	N = 196; grades 2–5; 65% male; 17% White; 58% Hispanic; 25% Black	Any asthma diagnosis	Hospital: control = 0.3, intervention = 0.3	Not reported	Texas
Kattan et al (15), 2006	Achievement of guidelines-based medical management	Computer-generated letters to medical providers	N = 937; aged 5–11 y; 62% male; 40% Hispanic, 40% Black, 7% White	Moderate to severe	ED: control = 3.0, intervention = 3.0	Not reported	7 US urban areas
Szefler et al (28), 2008^d^	Achievement of guidelines-based medical management	Asthma management program (treatment based on NAEPP guidelines)	N = 546; aged 12–20 y; 53% male; 64% Black, 23% Hispanic, 14% Other or mixed	Persistent	Hospital: control = not reported, intervention = 0.2	2004–2005	Ten centers (multi-city)
Teach et al (27), 2006	Education on asthma self-management, achievement of guidelines-based medical management, home visits, and linkages	Follow-up clinic visit (asthma self-monitoring and management, environmental modification, linkages, and referrals)	N = 488; aged 1–17 y; 63.9% male; 86% Black, 9% Hispanic	16% Severe persistent, 14% moderate persistent	ED: control = 2.4, intervention = 2.7	2002–2004	Children’s National Medical Center, Washington, DC
Wilson et al (29), 2001	Extinguishing smoking	ETS exposure-reduction (behaviorally based counseling sessions)	N = 87; aged 3–12 y; 51% male; 36% White, 38% Black, 44% Hispanic	43% moderate, 44% mild	Not reported (ED and hospital)	Not reported	Valley Children’s Hospital, Fresno County, California

Abbreviation: ED, emergency department; ETS, environmental tobacco smoke; NAEPP, National Asthma Education and Prevention Program; RCT, randomized controlled trial.

a EXHALE comprises a set of 6 strategies that contribute to asthma control: 1) education on asthma self-management, 2) extinguishing smoking and exposure to secondhand smoke, 3) home visits for trigger reduction and asthma self-management education, 4) achievement of guidelines-based medical management, 5) linkages and coordination of care across settings, and 6) environmental practices to reduce asthma triggers from indoor, outdoor, or occupational settings (4).

b Estimated incidence rate (per person per year).

c Study population included adults and children.

d Compared baseline rates with post-study rates for a single intervention arm.

### Example 1: Number of ED visits prevented

Ten RCTs satisfied our inclusion criteria for ED visits among children with asthma (Table 3A). Although each RCT evaluated an EXHALE strategy, the interventions described differed in the number of sessions, setting, population studied and overall content. 

**Table 3A:** Projected Number of Asthma-Related ED Visits Prevented in Population of Interest (Children With Asthma), by RCT Study and Intervention Type, Assuming 20,540^a^ ED Visits Annually Before Program Implementation

EXHALE strategy^b^	RCT study	Estimated effect, $RR_{RCT_{X}}\;$ (95% CI)^c^	Proportion of population of interest where intervention was offered, *Pr* d	Estimated no. of ED visits prevented by intervention^e^
Education on asthma self-management	Brown et al (21), 2006	0.60 (0.54 to 0.66)	0.20	1,643 (1,397 to 1,890)
	Bruzzese et al (20), 2011	0.47 (0.45 to 0.49)	0.20	2,177 (2,095 to 2259)
	Cowie et al (23), 2002	0.61 (0.51 to 0.72)	0.20	1,602 (1,150 to 2,013)
	Farber et al (24), 2004	0.88 (0.56 to 1.36)	0.20	493 (−1,479 to 1,808)
	Gorelick et al (25), 2006	0.97 (0.85 to 1.10)	0.20	123 (−411 to 616)
Achievement of guidelines-based medical management	Bender et al (11), 2015	1.50 (1.14 to 1.98)	0.15	−1,541^f^ (−3,019 to 431)
	Kattan et al (15), 2006	0.76 (0.75 to 0.77)	0.15	739 (709 to 770)
	Cabana and Thyne (22), 2006	0.69 (0.66 to 0.72)	0.15	955 (863 to 1,048)
Extinguishing smoking	Wilson et al (29), 2001	0.64 (0.50 to 0.81)	0.07	518 (273 to 719)
Education on asthma self-management, achievement of guidelines-based medical management, home visits, and linkages	Teach et al (27), 2006	0.47 (0.46 to 0.48)	0.025	272 (267 to 277)

Abbreviations: ED, emergency department; RCT, randomized control trial; RR, rate ratio.

a Estimated number from Los Angeles County, California (19).

b EXHALE comprises 6 strategies that contribute to asthma control: 1) education on asthma self-management, 2) extinguishing smoking and exposure to secondhand smoke, 3) home visits for trigger reduction and asthma self-management education, 4) achievement of guidelines-based medical management, 5) linkages and coordination of care across settings, and 6) environmental practices to reduce asthma triggers from indoor, outdoor, or occupational settings (4).

c RRs were calculated from group-specific (ie, control and intervention) ED rates provided by each RCT. For studies reporting risks, we estimated the group-specific rates before calculating the estimated study RR.

d Estimates are for illustrative purposes only. Parameter *Pr* depends on the scale of the intervention.

e Numbers in parentheses were calculated by using the upper and lower bound of the 95% CIs for $RR_{RCT_{X}}$.

f Negative number for prevented ED visits reflects an RR >1 and that the intervention was associated with an increase in ED visits.

On the basis of the effect estimate for strategy E, education on asthma self-management, the intervention described by Brown et al (21), for example, would have prevented an estimated 1,643 ED visits annually. For strategy A, in the intervention described by Kattan et al (15) would have prevented an estimated 729 ED visits per year. For strategy X, in the intervention described by Wilson et al, an estimated 518 ED visits would have been prevented annually (29).

### Example 2: Number of hospitalizations prevented

Eleven RCTs satisfied our criteria for hospitalizations among children with asthma (Table 3B). Each RCT had a unique set of features such as study population, demographic characteristics, distribution of asthma severity, setting, and content. On the basis of the effect estimate for strategy E, education on asthma self-management, the intervention described by Bruzzese et al (20), for example, would have prevented 417 hospitalizations annually. For strategy A, the intervention described by Szefler et al (28) would have prevented 293 hospitalizations annually; and for strategy X, the intervention described by Wilson et al (29) would have prevented 107 hospitalizations annually.

**Table 3B:** Projected Number of Asthma-Related Hospitalizations Prevented in Population of Interest (Children With Asthma), by RCT Study and Intervention Type, Assuming 2,639^a^ Hospitalizations Annually Before Program Implementation

EXHALE strategy^b^	RCT study	Estimated effect $RR_{RCT_{X}}$ (95% CI)^c^	Proportion of population of interest where intervention was offered, *Pr* d	Estimated no. of hospitalizations prevented by intervention^e^
Education on asthma self-management	Bruzzese et al (20), 2011	0.21 (0.14 to 0.30)	0.20	417 (369 to 454)
	Cowie et al (23), 2002	0.13 (0 to 15.78)^f^	0.20	459 (−7,801 to 528)
	Farber et al (24), 2004	5.95 (0.06 to 632.2)^f^	0.20	−2,613^g^ (−33,3147 to 496)
	Harish et al (13), 2001	1.63 (0.83 to 3.23)	0.20	−333^g^ (−1,177 to 90)
	Horner et al (14), 2016	0.41 (0.13 to 1.28)	0.20	311 (−148 to 459)
	Atherly et al (12), 2009	0.88 (0.26 to 2.99)	0.20	63 (−1,050 to 391)
Achievement of guidelines-based medical management	Bender et al (11), 2015	0.50 (0.21 to 1.19)	0.15	198 (−75 to 313)
	Szefler et al (28), 2008	0.26 (0.21 to 0.33)	0.15	293 (265 to 313)
	Halterman et al (26), 2004	0.31 (0.08 to 1.16)	0.15	273 (−63 to 364)
	Cabana and Thyne (22), 2006	0.73 (0.60 to 0.91)	0.15	107 (36 to 158)
Extinguishing smoking	Wilson et al (29), 2001	0.42 (0.16 to 1.07)	0.07	107 (−13 to 155)

a Estimated number of asthma hospitalizations in Los Angeles County, California (19).

b EXHALE comprises a set of 6 strategies that contribute to asthma control: 1) education on asthma self-management, 2) extinguishing smoking and exposure to secondhand smoke, 3) home visits for trigger reduction and asthma self-management education, 4) achievement of guidelines-based medical management, 5) linkages and coordination of care across settings, and 6) environmental practices to reduce asthma triggers from indoor, outdoor, or occupational settings (4).

c Rate ratios were calculated from group-specific (ie, control and intervention) ED rates provided by each RCT study. For studies reporting risks, we first estimated the group-specific rates before calculating the estimated study risk ratio.

d These estimates are for illustrative purposes only. Parameter *Pr* depends on the scale of the intervention.

e Numbers in parentheses were calculated by using the upper and lower bound of the 95% CIs for $RR_{RCT_{X}}$.

f Zero hospitalizations were reported in the intervention group in study by Cowie et al in 2002 (23) and for the control group in Farber et al in 2004 (24). For the $RR_{RCT_{X}}$ calculation, we used 0.5 hospitalizations for number of events for these groups (in place of zero).

g Negative number for prevented hospitalizations reflects an RR >1 and that the intervention was associated with an increase in hospitalizations.

## Discussion

We illustrated how to use effect estimates in the published literature to predict the effect of expanding access to interventions in the population of children with asthma. We demonstrated a practical method for converting effect estimates into rates of AAEs prevented by expanding EXHALE strategies. We used 15 recent RCTs to illustrate the method, but the method can also be applied to other study designs. These RCTs were conducted in various settings with different study populations, degrees of asthma severity, and intervention types. The treatment and control arms of RCTs are often not random samples of the population of interest. Public health practitioners can use the most appropriate RCT on the basis of considerations of comparability and transportability.

In the studies selected for our analysis, participants were evaluated to determine the effect of the program on rates of AAEs in treatment and control arms. However, the intervention might have already been available to some members of the study population as part of usual care. The comparison of the rates in the treatment and the control arms estimates the effect of the program compared with no program or usual care. Such a lack of compliance in an RCT or the number of dropouts in an observational study parallels the situation in the community where not all enrollees necessarily participated, received, or completed the intervention (30,31).

Two key challenges in using results of RCTs or other studies to estimate events prevented in a community are generalizability and transportability. Researchers distinguish between these 2 related concepts by using the term “generalizability” when study groups are selected from the community of interest and the term “transportability” when the study groups are not selected from the community of interest. In our context, transportability concerns the extent to which rates and effects from the selected studies can be used directly or with certain transport equations (32,33) to estimate effects in the community of interest. Pearl and Bareinboim argued in 2014 that the issues that determine whether and how to transport effect estimates are causal issues that reflect differences between populations and the mechanisms by which effects occur, rather than just differences in statistical distributions (32). In a theoretic approach and building on extensive work in causal inference, they formally represented causal relationships in “selection diagrams” and offered several transport formulas and guidance on when they apply to account for types of differences (32). Several other methods have recently been developed for transporting estimates from RCTs and other studies to a population of interest (33–36). These techniques often require information on certain covariate-specific effects (effect modifiers) and the covariate distribution in the population of interest. One study used in our analysis assessed effect modification and allowed us to illustrate our approach in that context (20).

Emphasizing practical considerations, Spiegelman et al argued that the choice of effect measure (eg, rate ratio or rate difference) for analyzing studies should be driven by model fit (16,18,37). They also cited anecdotal evidence to suggest that a rate ratio or risk ratio model might often fit well (16). They suggested transforming the effect measure to the scale of public health interest and standardizing to the distribution of risk factors (effect modifiers) in the population of interest. They cautioned that the required distributions of risk factors could be unavailable, consistent with the paucity of such information that we found.

Consistent with Spiegelman et al (18), we used a ratio scale for effect estimates. The validity of our estimates depends on the assumption that the causal effect in the population of interest, when measured on the ratio scale ($Rate_{P}/Rate_{\overset{-}{P}}^{*}$), is reasonably approximated by the effect estimate ${RR}_{RCT_{E}}$ from an RCT. Considerations for selection of studies that could make the assumption more plausible include the following: evaluation of the same intervention, similarity of the study population and community of interest with respect to baseline levels of asthma intervention, asthma severity, access to care, socioeconomic levels, health insurance, and demographic characteristics. One should also consider the existence and distribution of important effect modifiers. Similarity of baseline rates might also be considered, but it is not necessary if effects are not heterogeneous on the chosen scale (eg, ratio).

### Limitations

We found only 1 RCT for strategy X (29) and no RCTs met our selection criteria separately for strategy H or for strategy E2. Furthermore, certain combinations of strategies have been recommended. For example, strategies A and E1 together and A, E1, and H together were recommended as part of a stepwise approach for children according to levels of asthma control (38), but we found only 1 RCT for the strategies A and E1 together, making it difficult to evaluate the effect of this combination (27). In addition, the characteristics of the study populations in the RCTs included in our analysis differed in terms of race and ethnicity, age, asthma severity, and urbanicity. Some populations were not well represented in the selected RCTs. For example, only 1 RCT (14) focused on a rural population and only one (21) evaluated effect modification, which can be important for estimating effects in the community. Although we illustrated our methods by using RCTs, our approach can be used with other study designs that provide valid effect estimates for these strategies, combinations of strategies, effect modifications, and population subgroups.

### Conclusion

Expanding access to the evidence-based EXHALE strategies can contribute to reducing the number of asthma-related ED visits and hospitalizations. The method we illustrated draws on the best available evidence from RCTs to estimate effects on rates of AAEs in a community that result from expanding access to an EXHALE strategy. This method can be used to calculate the anticipated effects of future asthma interventions or to calculate the effects of programs that have been implemented. Public health practitioners can use the method outlined in this article and the current base of RCT literature in their planning and evaluation of interventions toward the overall goal of reducing the number of asthma deaths.

## References

[1] Miller GF , Coffield E , Leroy Z , Wallin R . Prevalence and costs of five chronic conditions in children. J Sch Nurs 2016;32(5):357–64. 10.1177/1059840516641190 27044668 PMC5010981

[2] Centers for Disease Control and Prevention. Most recent national asthma data. 2020. Last reviewed December 13, 2022. Accessed October 3, 2022. https://www.cdc.gov/asthma/most_recent_national_asthma_data.htm

[3] Sullivan PW , Ghushchyan V , Navaratnam P , Friedman HS , Kavati A , Ortiz B , The national cost of asthma among school-aged children in the United States. Ann Allergy Asthma Immunol 2017;119(3):246–252.e1. 10.1016/j.anai.2017.07.002 28890020

[4] Centers for Disease Control and Prevention. EXHALE. A technical package to control asthma. 2018. Page last reviewed July 6, 2021. Accessed October 3, 2022. https://www.cdc.gov/asthma/pdfs/EXHALE_technical_package-508.pdf

[5] Centers for Disease Control and Prevention. Controlling Childhood Asthma and Reducing Emergencies (CCARE). 2019. Last reviewed December 12, 2022. Accessed May 1, 2022. https://www.cdc.gov/asthma/ccare.htm

[6] Crocker DD , Kinyota S , Dumitru GG , Ligon CB , Herman EJ , Ferdinands JM , ; Task Force on Community Preventive Services. Effectiveness of home-based, multi-trigger, multicomponent interventions with an environmental focus for reducing asthma morbidity: a community guide systematic review. Am J Prev Med 2011;41(2 Suppl 1):S5–32. 10.1016/j.amepre.2011.05.012 21767736

[7] Zahran HS , Bailey CM , Damon SA , Garbe PL , Breysse PN . Vital signs: asthma in children — United States, 2001–2016. MMWR Morb Mortal Wkly Rep 2018;67(5):149–55. 10.15585/mmwr.mm6705e1 29420459 PMC5812476

[8] Boyd M , Lasserson TJ , McKean MC , Gibson PG , Ducharme FM , Haby M . Interventions for educating children who are at risk of asthma-related emergency department attendance. Cochrane Database Syst Rev 2009;2009(2):CD001290. 10.1002/14651858.CD001290.pub2 19370563 PMC7079713

[9] Okelo SO , Butz AM , Sharma R , Diette GB , Pitts SI , King TM , Interventions to modify health care provider adherence to asthma guidelines: a systematic review. Pediatrics 2013;132(3):517–34. 10.1542/peds.2013-0779 23979092 PMC4079294

[10] Harris K , Kneale D , Lasserson TJ , McDonald VM , Grigg J , Thomas J . School-based self-management interventions for asthma in children and adolescents: a mixed methods systematic review. Cochrane Database Syst Rev 2019;1(1):CD011651. 10.1002/14651858.CD011651.pub2 30687940 PMC6353176

[11] Bender BG , Cvietusa PJ , Goodrich GK , Lowe R , Nuanes HA , Rand C , Pragmatic trial of health care technologies to improve adherence to pediatric asthma treatment: a randomized clinical trial. JAMA Pediatr 2015;169(4):317–23. 10.1001/jamapediatrics.2014.3280 25664620

[12] Atherly A , Nurmagambetov T , Williams S , Griffith M . An economic evaluation of the school-based “power breathing” asthma program. J Asthma 2009;46(6):596–9. 10.1080/02770900903006257 19657901

[13] Harish Z , Bregante AC , Morgan C , Fann CS , Callaghan CM , Witt MA , A comprehensive inner-city asthma program reduces hospital and emergency room utilization. Ann Allergy Asthma Immunol 2001;86(2):185–9. 10.1016/S1081-1206(10)62689-0 11258688

[14] Horner SD , Brown A , Brown SA , Rew DL . Enhancing asthma self-management in rural school-aged children: a randomized controlled trial. J Rural Health 2016;32(3):260–8. 10.1111/jrh.12150 26431213 PMC4818726

[15] Kattan M , Crain EF , Steinbach S , Visness CM , Walter M , Stout JW , A randomized clinical trial of clinician feedback to improve quality of care for inner-city children with asthma. Pediatrics 2006;117(6):e1095–103. 10.1542/peds.2005-2160 16740812

[16] Spiegelman D , VanderWeele TJ . Evaluating public health interventions: 6. modeling ratios or differences? Let the data tell us. Am J Public Health 2017;107(7):1087–91. 10.2105/AJPH.2017.303810 28590865 PMC5463222

[17] Lash TL , VanderWeele TJ , Haneuse S , Rothman KJ . Modern epidemiology, 4th ed. Wolters Kluwer; 2021. 10.1007/s10654-021-00778-w PMC8416883 34216355

[18] Spiegelman D , Khudyakov P , Wang M , Vanderweele TJ . Evaluating public health interventions: 7. Let the subject matter choose the effect measure: ratio, difference, or something else entirely. Am J Public Health 2018;108(1):73–6. 10.2105/AJPH.2017.304105 29161073 PMC5719681

[19] California Department of Public Health. Asthma hospitalization rates by county — datasets. Accessed December 23, 2021. https://data.chhs.ca.gov/dataset/asthma-emergency-department-visit-rates

[20] Bruzzese JM , Sheares BJ , Vincent EJ , Du Y , Sadeghi H , Levison MJ , Effects of a school-based intervention for urban adolescents with asthma. A controlled trial. Am J Respir Crit Care Med 2011;183(8):998–1006. 10.1164/rccm.201003-0429OC 21139088 PMC3086747

[21] Brown MD , Reeves MJ , Meyerson K , Korzeniewski SJ . Randomized trial of a comprehensive asthma education program after an emergency department visit. Ann Allergy Asthma Immunol 2006;97(1):44–51. 10.1016/S1081-1206(10)61368-3 16892780

[22] Cabana MD , Thyne SM . An emergency department-based follow-up clinic can improve asthma outcomes. J Pediatr 2006;149(5):725. 10.1016/j.jpeds.2006.08.035 17095354

[23] Cowie RL , Underwood MF , Little CB , Mitchell I , Spier S , Ford GT . Asthma in adolescents: a randomized, controlled trial of an asthma program for adolescents and young adults with severe asthma. Can Respir J 2002;9(4):253–9. 10.1155/2002/106262 12195271

[24] Farber HJ , Chi FW , Capra A , Jensvold NG , Finkelstein JA , Lozano P , Use of asthma medication dispensing patterns to predict risk of adverse health outcomes: a study of Medicaid-insured children in managed care programs. Ann Allergy Asthma Immunol 2004;92(3):319–28. 10.1016/S1081-1206(10)61569-4 15049395

[25] Gorelick MH , Meurer JR , Walsh-Kelly CM , Brousseau DC , Grabowski L , Cohn J , Emergency department allies: a controlled trial of two emergency department-based follow-up interventions to improve asthma outcomes in children. Pediatrics 2006;117(4 Pt 2 Supplement_2):S127–34. 10.1542/peds.2005-2000J 16777828

[26] Halterman JS , Szilagyi PG , Yoos HL , Conn KM , Kaczorowski JM , Holzhauer RJ , Benefits of a school-based asthma treatment program in the absence of secondhand smoke exposure: results of a randomized clinical trial. Arch Pediatr Adolesc Med 2004;158(5):460–7. 10.1001/archpedi.158.5.460 15123479

[27] Teach SJ , Crain EF , Quint DM , Hylan ML , Joseph JG . Improved asthma outcomes in a high-morbidity pediatric population: results of an emergency department-based randomized clinical trial. Arch Pediatr Adolesc Med 2006;160(5):535–41. 10.1001/archpedi.160.5.535 16651498

[28] Szefler SJ , Mitchell H , Sorkness CA , Gergen PJ , O’Connor GT , Morgan WJ , Management of asthma based on exhaled nitric oxide in addition to guideline-based treatment for inner-city adolescents and young adults: a randomised controlled trial. Lancet 2008;372(9643):1065–72. 10.1016/S0140-6736(08)61448-8 18805335 PMC2610850

[29] Wilson SR , Yamada EG , Sudhakar R , Roberto L , Mannino D , Mejia C , A controlled trial of an environmental tobacco smoke reduction intervention in low-income children with asthma. Chest 2001;120(5):1709–22. 10.1378/chest.120.5.1709 11713157

[30] Chêne G , Morlat P , Leport C , Hafner R , Dequae L , Charreau I , Intention-to-treat vs. on-treatment analyses of clinical trial data: experience from a study of pyrimethamine in the primary prophylaxis of toxoplasmosis in HIV-infected patients. ANRS 005/ACTG 154 Trial Group. Control Clin Trials 1998;19(3):233–48. 10.1016/S0197-2456(97)00145-1 9620807

[31] Feinman RD . Intention-to-treat. What is the question? Nutr Metab (Lond) 2009;6(1):1. 10.1186/1743-7075-6-1 19134186 PMC2631586

[32] Pearl J , Bareinboim E . External validity: from do-calculus to transportability across populations. Stat Sci 2014;29(4):579–95. 10.1214/14-STS486

[33] Westreich D , Edwards JK , Lesko CR , Stuart E , Cole SR . Transportability of trial results using inverse odds of sampling weights. Am J Epidemiol 2017;186(8):1010–4. 10.1093/aje/kwx164 28535275 PMC5860052

[34] Dahabreh IJ , Robertson SE , Steingrimsson JA , Stuart EA , Hernán MA . Extending inferences from a randomized trial to a new target population. Stat Med 2020;39(14):1999–2014. 10.1002/sim.8426 32253789

[35] Mumford SL , Schisterman EF . New methods for generalizability and transportability: the new norm. Eur J Epidemiol 2019;34(8):723–4. 10.1007/s10654-019-00532-3 31175532 PMC7994943

[36] Hong J-L , Webster-Clark M , Jonsson Funk M , Stürmer T , Dempster SE , Cole SR , Comparison of methods to generalize randomized clinical trial results without individual-level data for the target population. Am J Epidemiol 2019;188(2):426–37. 10.1093/aje/kwy233 30312378 PMC6357813

[37] Spiegelman D , Zhou X . Evaluating public health interventions: 8. Causal inference for time-invariant interventions. Am J Public Health 2018;108(9):1187–90. 10.2105/AJPH.2018.304530 30024804 PMC6085031

[38] Centers for Disease Control and Prevention. CDC’s 6/18 Initiative: accelerating evidence into action. 2021. Accessed April 22, 2022. https://www.cdc.gov/sixeighteen

